# Identifying barriers and facilitators to successful implementation of computerized clinical decision support systems in hospitals: a NASSS framework-informed scoping review

**DOI:** 10.1186/s13012-023-01287-y

**Published:** 2023-07-26

**Authors:** Bridget Abell, Sundresan Naicker, David Rodwell, Thomasina Donovan, Amina Tariq, Melissa Baysari, Robin Blythe, Rex Parsons, Steven M. McPhail

**Affiliations:** 1 Australian Centre for Health Services Innovation and Centre for Healthcare Transformation, School of Public Health and Social Work, Faculty of Health, Queensland University of Technology, Brisbane, QLD Australia; 2 Biomedical Informatics and Digital Health, School of Medical Sciences, Faculty of Medicine and Health, The University of Sydney, Camperdown, Australia

**Keywords:** Barriers and facilitators, Implementation science, CDSS, Clinical decision support systems, Hospital, Digital health, NASSS

## Abstract

**Background:**

Successful implementation and utilization of Computerized Clinical Decision Support Systems (CDSS) in hospitals is complex and challenging. Implementation science, and in particular the Nonadoption, Abandonment, Scale-up, Spread and Sustainability (NASSS) framework, may offer a systematic approach for identifying and addressing these challenges. This review aimed to identify, categorize, and describe barriers and facilitators to CDSS implementation in hospital settings and map them to the NASSS framework. Exploring the applicability of the NASSS framework to CDSS implementation was a secondary aim.

**Methods:**

Electronic database searches were conducted (21 July 2020; updated 5 April 2022) in Ovid MEDLINE, Embase, Scopus, PyscInfo, and CINAHL. Original research studies reporting on measured or perceived barriers and/or facilitators to implementation and adoption of CDSS in hospital settings, or attitudes of healthcare professionals towards CDSS were included. Articles with a primary focus on CDSS development were excluded. No language or date restrictions were applied. We used qualitative content analysis to identify determinants and organize them into higher-order themes, which were then reflexively mapped to the NASSS framework.

**Results:**

Forty-four publications were included. These comprised a range of study designs, geographic locations, participants, technology types, CDSS functions, and clinical contexts of implementation. A total of 227 individual barriers and 130 individual facilitators were identified across the included studies. The most commonly reported influences on implementation were fit of CDSS with workflows (19 studies), the usefulness of the CDSS output in practice (17 studies), CDSS technical dependencies and design (16 studies), trust of users in the CDSS input data and evidence base (15 studies), and the contextual fit of the CDSS with the user’s role or clinical setting (14 studies). Most determinants could be appropriately categorized into domains of the NASSS framework with barriers and facilitators in the “Technology,” “Organization,” and “Adopters” domains most frequently reported. No determinants were assigned to the “Embedding and Adaptation Over Time” domain.

**Conclusions:**

This review identified the most common determinants which could be targeted for modification to either remove barriers or facilitate the adoption and use of CDSS within hospitals. Greater adoption of implementation theory should be encouraged to support CDSS implementation.

**Supplementary Information:**

The online version contains supplementary material available at 10.1186/s13012-023-01287-y.

Contributions to the literature

- To our knowledge, this is the first review to systematically identify, classify, and map peer-reviewed literature about barriers and facilitators of hospital-based CDSS implementation using an implementation science framework.
- This review supports the value of the NASSS framework for assessing context and guiding CDSS implementation.
- This review identified a need for CDSS to be designed to fit the workflows and contexts of clinical practice for effective implementation outcomes.
- No determinants mapped to the “embedding over time” domain of NASSS, underscoring the need to examine how implementation factors change over time.

## Background

Clinical Decision Support Systems (CDSS) are digital tools that can assist healthcare providers in making clinical decisions by providing targeted information [1]. They have the potential to improve various aspects of healthcare delivery, such as patient safety, clinical management, diagnostic support, cost management, and administrative efficiency [2]. CDSS can take different forms, such as guideline-based algorithms for chronic disease risk and screening, personalized medication dosing, and alerts for potential or historical adverse events [3, 4].

Despite the varied applications and benefits of CDSS, there are challenges related to their adoption, appropriate use, and sustainment over time in clinical settings [5, 6]. Challenges include poor uptake, inappropriate use, unintended consequences, and abandonment of the technology over time [5, 7–9]. The reasons for these challenges are multifactorial and include the social and organizational complexity of large health systems which makes it difficult to plan for downstream consequences with confidence [10, 11]. CDSS implementation is also affected by unforeseen system modifications to adapt to fast changing contexts [12]. Ideally, hospital-system and research stakeholders need to identify constructs that have been shown to influence implementation processes and outcomes, understand potential mechanisms of change, and identify potential barriers and facilitators to successful implementation [13, 14]. This is important as contextually informed implementation strategies targeting clinician behavior are a stronger predictor of CDSS uptake than technological design and content features [6]. Consequently, evidence-based guidance to inform and plan for such contextually appropriate implementation is necessary for greater systematic success of technological interventions in healthcare systems [5–12].

Implementation science offers theory-informed systematic processes for research translation [15]. Its theories, models, and frameworks help to identify barriers and facilitators throughout the implementation process and at different contextual levels. This includes both the immediate context of individuals receiving health-related interventions, their healthcare providers, healthcare organizations, and other stakeholders involved in care delivery, as well as the broader political, economic, and social setting [16]. Yet, few previous reviews of CDSS have applied a comprehensive implementation science framework within the context of the implementation processes [2, 3, 6].

The Nonadoption, Abandonment, Scale-up, Spread and Sustainability (NASSS) framework was developed to understand the successful implementation of technology-supported health (and social) care programs [17]. It positions technological interventions as part of a complex system [18] and has been used to guide implementation efforts and identify factors that influence implementation success in digital health [19–21]. A previous systematic review used the NASSS framework to assess contextual factors that can influence implementation success of virtual care in primary care settings [22]. The authors concluded that applying the NASSS framework helped to identify key contextual factors that can influence implementation success.

There is a substantial amount of literature which appears to examine different aspects of the implementation process for CDSSs within hospitals [3, 23, 24]. However, there have been limited attempts to classify these findings according to a deterministic implementation framework like NASSS. Such an approach may identify generalizable determinants to scale CDSS implementation efforts across varying hospital settings, integrate implementation processes into a unified framework, and inform more synergetic and comparative CDSS implementation research moving forward.

This study aimed to apply a qualitative reflexive analysis to identify, classify, and describe the potential barriers and facilitators, identified within peer-reviewed literature, that influence the implementation process (adoption, uptake, and use over time) of CDSS in hospital settings and map these findings to the NASSS framework. A secondary aim was to explore the applicability of the NASSS framework to CDSS implementation within a hospital context.

## Method

A scoping review methodology was deemed appropriate to address the aims of this study as our goal was not to evaluate the evidence, but to identify and map key determinants of CDSS implementation and identify knowledge gaps [25]. This scoping review was conducted in accordance with the recommendations of Arksey and O’Malley [26] and Levac, Colquhoun [27]. Step (1) involved identifying the research question as “What are the barriers and facilitators to implementation of CDSS in hospital settings,” with the sub-question of “How can the determinants of CDSS implementation in hospital settings be categorized using an implementation science framework.” The remaining review steps consisted of (2) identifying relevant studies, (3) selecting studies, (4) charting the data, and (5) collating, summarizing, and reporting the results. The reporting of this scoping review was guided by the Preferred Reporting Items for Systematic Reviews and Meta-Analyses extension for Scoping Reviews (PRISMA-ScR) checklist [28]. A protocol for this review and mapping was developed by BA before commencing searches but was not published.

### Identifying relevant studies

We searched electronic databases on 21 July 2020 and again on 5 April 2022. The databases searched were Ovid MEDLINE (via EBSCOhost), Embase, Scopus, PyscInfo, and CINAHL (via EBSCOhost). The search strategy was iteratively created and refined using the SearchRefinery tool [29] with MeSH terms and keywords related to CDSS, implementation, and hospital settings (see Additional file 1). The final search string is provided in Table 1. The Polyglot Search Translator [30] was used to translate the Ovid MEDLINE string across all other databases, which were also searched for additional subject headings. The final search results were exported into EndNote [31], duplicates removed, and remaining records uploaded into Rayyan [32] for screening. Generation of the search terms, execution of both searches, and deduplication of records were all conducted by the same author (BA) in consultation with a medical librarian.

**Table 1:** Final search string used to identify articles for the study

Database	Search string
MEDLINE (Ovid)	((exp Decision Support Systems, Clinical/ OR Artificial Intelligence.ti,ab. OR Dashboard.ti,ab. OR Reminder system*.ti,ab. OR electronic feedback.ti,ab. OR CDSS.ti,ab.) AND (barrier*.ti,ab. OR Facilitat*.ti,ab.))

### Study selection

Inclusion and exclusion criteria were determined prior to the database searches. For inclusion, articles were required to be (1) published, peer-reviewed original research studies of qualitative, quantitative or mixed methods design, (2) prospectively or retrospectively reported on measured or perceived barriers and/or facilitators to implementation and adoption of CDSS, (3) based in hospital settings. Studies of similar and potentially overlapping concepts, including (4) attitudes of healthcare professionals towards CDSS, were also included. All health conditions, hospital settings (inpatient and outpatient), and patient groups were eligible.

For this review CDSS was defined as any technology system designed to provide health professionals or operational staff with information/decision support that was filtered or targeted to a specific user, patient, or situation [33]. All decision support typologies, including both knowledge-based and non-knowledge-based systems, were included. There were no exclusion criteria related to the ways by which the users engaged with the decision support information; for example, dashboards, interruptive alerts, or reminder systems were all considered within scope.

Articles were excluded if they focused on consumer/patient facing decision support, e.g., decision aids, or primarily on CDSS development and not implementation. No language or date restrictions were applied, but papers unobtainable in full-text format (e.g., conference abstracts) were excluded.

Preliminary screening was piloted by all reviewers with a small sample of records to improve consistency of study selection criteria. The titles and abstracts of all records were then independently co-screened in Rayyan by eight reviewers working in pairs. Coding pairs met with a third reviewer (BA) to reach consensus about disagreements. Records that proceeded to full-text review were obtained and independently co-screened by two reviewer pairs (BA and SN, TD and DR). Disagreements about the inclusion of articles between reviewers in each pair were discussed in meetings facilitated by a third reviewer from the other pair until consensus was reached. As recommended by Levac, Colquhoun, and O’Brien [27], the core multidisciplinary review team (BA, SN, TD, and DR) met regularly to discuss any uncertainties related to study selection or review scope.

### Charting the data

A shared online data extraction form was created using Microsoft Excel and was piloted and refined with several included studies. Subsequently, the following information was extracted from each included article independently by two authors (TD, DR): study identifiers (e.g., year, author, country), study type (e.g., observational, RCT), research methodology (e.g., quantitative, qualitative, mixed methods), participant descriptors (e.g., sample size, professional discipline), description of CDSS, clinical or organizational setting of CDSS (e.g., ward, hospital or multiple sites), key barriers to implementation/adoption, key facilitators to implementation/adoption, and any other details noted as potentially important for the study (e.g. implementation processes or frameworks). To address discrepancies in the extracted data, reporting issues, and data verification, three authors (TD, DR, SN) held meetings and iteratively updated the extraction form. In these cases, the relevant manuscript was thoroughly re-examined, specifically referring to each variable in the extraction document, until a consensus was reached.

### Collating, reporting, and summarizing the results

There were several steps in collating and reporting the results which were conducted iteratively in a series of day-long workshops attended by four of the authors (TD, DR, BA, SN).

#### Data analysis

Our approach involved basic qualitative content analysis of all included publications (qualitative, quantitative, and mixed methods) using open coding and allocation into higher-order categories [34]. First, using the data extraction Excel sheet, each barrier and facilitator was assigned a short summarizing phrase or word that meaningfully captured its essence. These initial codes were discussed and revised with some original codes being discarded, amended, or subsumed by other codes to create higher-order codes. Following this, the content of each higher-order code and the underlying meaning of each was examined and discussed. The outcome of this process was to reflexively map each of the higher-order codes onto one or more of the NASSS domains and sub-domains with which they aligned.

The analysis was primarily deductive with the intent was to align barriers and facilitators identified in the literature with pre-existing domains within the NASSS framework. It is important to note that, in the analysis, no restriction was placed on the number of domains or sub-domains with which an individual barrier or facilitator could align. This was because the object of the study was not to strictly categorize barriers and facilitators using the NASSS but to understand the role of such a theoretical framework in capturing the complexity of CDSS implementation and adoption within real-world hospital systems.

A descriptive numerical summary of all included studies, and the individual barriers and facilitators to CDSS implementation was collated. We also developed a visual matrix including each study’s barriers and facilitators mapped across NASSS domains to demonstrate common findings and identify gaps in the research. Finally, a tree map was created to visualize the most frequently reported determinants to CDSS implementation and adoption in the included studies.

## Results

**Fig. 1:**
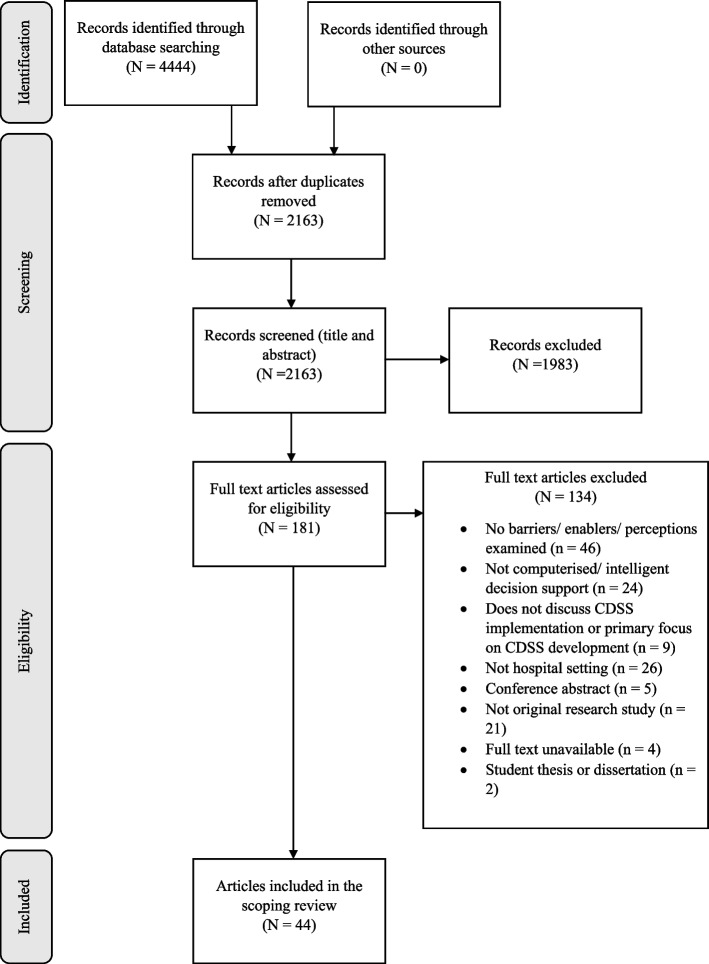
Preferred Reporting Items for Systematic Reviews and Meta-Analyses flow diagram for article selection

After deduplication, 2163 articles were identified in the database searches. Based on title and abstract screening, 1983 of these were excluded. The remaining 181 full-text publications were retrieved and assessed for eligibility, resulting in the exclusion of 137 studies. The 44 remaining publications were included in this scoping review. The article selection process and reasons for exclusion are presented in Fig. 1.

### Study characteristics

The characteristics of the 44 included studies, including their aim, participants, and CDSS type, are presented in Table 2. The studies were published between 2002 and 2022 with approximately 70% published since 2015. Most studies were published by authors residing in North America (*n* = 24). There were a mix of study designs, although most employed qualitative or mixed methods (*n* = 38). Ten studies were conducted as a precursor to CDSS implementation with the remainder completed during (peri) or post-implementation. CDSS were implemented across a wide range of clinical contexts and conditions (e.g., pulmonary embolism), uses (e.g., antimicrobial stewardship), patient cohorts (e.g., older persons), or combinations of these (e.g., imaging for pediatric traumatic brain injury). However, over one quarter of studies (*n* = 12) focused on CDSS intended for use in hospital emergency departments. Participants in the studies were overwhelmingly those engaged in clinical or patient-facing roles (e.g., physicians, nurses, pharmacists) with almost all studies (*n* = 40) clearly identifying individuals occupying these roles as participants.

**Table 2:** Characteristics of studies included in the scoping review and evidence map

Author, year, and country of study	Study design	Brief study aim, objective, or focus	Description of CDSS implemented	Participants in study
Barriers and facilitators assessed pre-implementation				
Blanco et al., 2018, USA [35]	Qual	Understand healthcare workers perceptions of barriers and facilitators related to the uptake of computerized tools to reduce Clostridium difficile infection.	EHR-integrated CDSS automatically generates an order for contact precautions when Clostridium difficile testing is ordered.	Nurses, physicians, physician assistants, pharmacists, radiologist technicians, and environmental service workers (*n* = 47).
Flynn et al., 2015, UK [36]	Qual	Develop a CDSS to support patient-specific clinical decision-making related to thrombolysis for acute ischaemic stroke and communicate personalized risk information to patients and families.	Computerized decision Aid for Stroke thrombolysis (COMPASS); provides decision support for thrombolysis.	Stroke physicians, ED physicians, and stroke nurse practitioners (*n* = 31).
Hasnie et al., 2018, USA [37]	Mixed methods	Develop a CDSS to provide guidance for clinicians at the point of care when treating patients with familial hypercholesterolemia.	Not yet developed.	Specialists including cardiologists, endocrinologists, medical geneticists, and primary care physicians (*n* = 210 survey, *n* = 19 focus groups).
Laka et al., 2021, Australia [7]	Mixed methods	Identify individual, organizational and system-level factors that influence and moderate the adoption, use and perceptions of use of a CDSS for antimicrobial management.	Connects evidence-based information on antibiotic prescribing with patient information to present accurate, real-time information to assist clinical decision-making.	Antibiotic prescribing clinicians in hospitals and primary care (*n* = 180).
Melnick et al., 2019, USA [38]	Qual	Describe challenges to implementation and scalability of CDSS for ED-initiated interventions for opioid use disorder.	EHR-integrated web-based CDSS providing optional assessment tools and treatment pathways for initiating buprenorphine treatment in the ED.	ED Physicians (*n* = 26).
Mugabe, 2021, New Zealand [39]	Quant	Investigate the barriers, facilitators and likelihood of radiation therapy professionals adopting AI to aid treatment planning.	CDSS not specified; focused on AI and machine learning to improve standards of care, reduce side effects, and enhance quality of life.	Clinicians and specialists involved in radiation treatment (*n* = 101).
Ploegmakers et al., 2022, Europe [40]	Quant	Assess barriers and facilitators for CDSS use by physicians treating community-dwelling and hospitalized older patients at risk of falls.	EMR-integrated to support clinicians in medication reviews and deprescribing decisions regarding fall-risk-inducing drugs in older falls patients.	Primary, secondary, and tertiary care physicians, nurse practitioners, and physicians’ assistants across Europe (*n* = 581).
Westafer et al., 2020, USA [41]	Qual	Identify barriers and facilitators to the adoption of evidence-based diagnostic testing for Pulmonary Embolism in the ED.	Risk stratification CDSS tool utilizing scores from recognized clinical guidelines.	Physicians from twelve academic and community hospitals (*n* = 23).
Yadav et al., 2015, USA [42]	Mixed methods	Use human factors engineering methods to design a more appropriate CDSS for pediatric trauma resuscitations.	Real-time rule-based CDSS based on dichotomous (yes/no) responses to questions entered by the clinician.	Pediatric emergency medicine providers (*n* = 40), human factors engineers (*n* = 3), emergency medicine academic faculty members (*n* = 5).
Barriers and facilitators assessed during or post-implementation				
Ballard et al., 2016, USA [43]	Quant	Assess clinician adoption of CDSS for site-of-care decision-making for ED patients with acute pulmonary embolism and characteristics associated with increased likelihood of use.	EHR linked assistive CDSS calculates the Pulmonary Embolism Severity Index (PESI) score, and provides PESI stratification data and risk profiles, and a list of additional reasons to consider admission of patients.	ED staff, *n* not clearly stated.
Bersani et al., 2020, USA [44]	Mixed methods	Conduct implementation evaluation of CDSS dashboard for patient safety including use, and perceptions of its usability and effectiveness.	Dashboard utilizing real-time EHR data to assist clinicians to quickly assess high-priority safety domains for patients admitted to hospital.	Nursing staff, prescribers, unit leadership, and other staff (*n* = 413).
Bowen et al., 2011, Canada [45]	Mixed	Assess effectiveness of incorporating guidelines into a CPOE for pediatric diagnostic imaging, identifying implementation barriers, facilitators and key stakeholders’ perspectives.	Compares diagnostic imaging orders with clinical guidelines and suggests alternative more appropriate order.	Physicians (*n* = 104).
Campion et al., 2011, USA [46]	Qual	Highlight barriers and facilitators to CDSS use for intensive insulin therapy by nurses in intensive care units.	A dosing calculation embedded in the CPOE.	Surgical ICU and trauma ICU nurses (49 h of observation).
Chow et al., 2014, Singapore [47]	Mixed methods	Understand physicians’ perceptions of a hospital’s antibiotic CDSS and the impact of psychosocial factors on acceptance of treatment recommendations.	CDSS integrates AMS with electronic prescribing.	Senior and junior doctors (*n* = 265 survey, *n* = 11 focus groups).
Chua et al., 2018, Singapore [48]	Qual	Explore hospital physicians’ perceptions and attitudes towards antimicrobial stewardship programs and CDSS.	CDSS provides patient-specific evidence-based antibiotic recommendations at the point of prescribing.	Senior and junior hospital physicians (*n* = 37).
Chung et al., 2017, USA [49]	Qual	Determine cultural beliefs, barriers and facilitators to implementation of EHR-integrated CDSS for antimicrobial stewardship in the pediatric ED.	Yet to be developed.	Bioinformatics, quality and safety, medical leaders in AMS, pediatric emergency medicine, pediatricians, clinical decision support teams, physician assistants, and nurse practitioners (*n* = 5 interviews, *n* = 17 focus groups).
Collins et al., 2012, Ireland [50]	Mixed	Inform user-centered design of a CDSS for oncology, and understand attitudes and knowledge of physicians and pharmacists about CDSS in oncology.	CDSS makes clinical suggestions based on the information submitted by the user or using evidence-based medicine.	Medical oncologists and oncology pharmacists (*n* = 41).
Cresswell et al., 2017, UK [10]	Qual	Explore how healthcare workers and organizations accommodated the introduction of CPOE and CDSS for hospital prescribing over time.	CPOE and CDSS for hospital prescribing which varied in type of system implemented (e.g., stand-alone vs EHR-integrated).	Physicians, nurses, pharmacists, allied health professionals, support staff, implementation managers, technicians, and system vendors across six hospitals (*n* = 173 interviews, *n* = 24 observations, *n* = 17 document audits).
de Vries et al., 2013, the Netherlands [51]	Quant	Explore perceived barriers and differences of heart failure nurses and cardiologists to using a CDSS for heart failure treatment, and assess relevance and influence of knowledge management.	A specific CDSS in the treatment of heart failure patients but is not described.	Cardiologists and heart failure nurses (*n* = 162).
English et al., 2017, USA [52]	Quant	Determine clinical pharmacists’ acceptance of a CDSS for surveillance of pharmaceutical therapies across a health system.	CDSS provides real-time surveillance of pharmaceutical therapies in a dashboard view. Includes rules against medication, laboratory, and demographic patient data.	Clinical pharmacists (*n* = 25).
Giuliano et al., 2018, USA [53]	Qual	Explore process of pharmacists using a single CDSS tool to perform antimicrobial stewardship across a healthcare system.	EHR-integrated CDSS identifies patients for prospective evaluation and intervene to improve antimicrobial prescribing.	Hospital-based pharmacists (*n* = 19).
Glassman et al., 2002, USA [54]	Quant	Understand benefits and barriers to use of automated, embedded CPOE drug alerts in outpatient care across a Veterans Affairs health care system.	EHR medication ordering program incorporating “critical” or “significant” automated alerts for approximately two thousand specified drug combinations.	Internal medicine (general and subspecialty), neurology, physical medicine, and psychiatry clinicians (*n* = 168).
Goud et al., 2010, the Netherlands [55]	Qual	Understand the effect of a CDSS on cognitive, organizational, and environmental barriers and facilitators which may impact cardiac rehabilitation guideline implementation.	Assists needs assessments for cardiac rehabilitation according to Dutch multidisciplinary cardiac rehabilitation guidelines.	Cardiac rehabilitation nurses (*n* = 29).
Grau et al., 2019, USA [56]	Qual	Understand barriers and facilitators to physician use of a smoking cessation CDSS and ascertain how to improve its uptake and use.	E-STOPS, an EHR embedded CDSS that alerts physicians of the patient’s smoking status and facilitates selection of evidence-based smoking cessation treatment options.	Physicians and internal medicine residents (*n* = 21).
Green et al., 2019, USA [57]	Mixed methods	Understand the factors that may limit use and perceived usefulness of medical calculators (including EHR integration).	Computer software medical calculators input patient supplied and/or clinically sourced data and returns a discrete answer through an equation, a decision tree/questionnaire, or an algorithm.	Physicians (*n* = 108).
Gutenstein et al., 2019, New Zealand [58]	Qual	Create a digital clinical pathway for acute chest pain in the ED by combining clinical workflow, decision support, documentation, and research within the EHR.	A digital clinical pathway that acts as a pragmatic guide and a map of clinical workflow that describes the patient journey through a local health system.	Emergency medicine and cardiology physicians (*n* = 10).
Jacobs et al., 2014, USA [59]	Mixed methods	Describe current clinical information system functionality for laboratory monitoring of immunosuppressive care, describe guideline use to enable computable logic and alerts to support guideline adherence, and explore CDS implementation barriers in liver transplant centers.	Yet to be developed.	Liver transplant care team members (*n* = 80). Surveys completed by one or more team members.
Johnson et al., 2015, UK [60]	Mixed methods	Understand factors influencing feasibility and impact on provider behavior of a CDSS to optimize angina management in chest pain clinics.	Web-based CDSS supports investigation and medication decisions for patients with onset stable chest pain.	Cardiologists, specialist cardiac nurses, physiologists (*n* = 74); and analysis of 285 patient records.
Lai et al., 2006, USA [61]	Mixed methods	Understand reasons underlying limited use of acute cardiac ischemia CDSS in ED, and explore potential of computer-based tutorial to overcome barriers to CDSS use.	The Acute Cardiac Ischemia-Time Insensitive Predictive Instrument is printed in real time on the ECG header.	Internal medicine residents (*n* = 16).
Lesselroth et al., 2011, USA [62]	Mixed methods	Describe a CDSS intervention to improve adherence of surgeons to DVT prophylaxis recommendations and use sociotechnical theory to identify new system adoption barriers and facilitators.	Provides surgeons with DVT prophylaxis recommendations aligning with clinical guidelines when accessing post-operative order sets.	Surgeons, *n* not clearly stated.
Liberati et al., 2017, Italy [12]	Qual	Examine perceived barriers and facilitators to CDSS uptake in hospitals at different stages of CDSS adoption, and construct a framework to guide CDSS implementation.	Commercial CDSS that compiles available evidence for treatment of a wide range of common conditions and provides point of care recommendations.	Doctors, nurses, managers, and IT specialists (*n* = 30).
Masterson et al., 2018, USA [63]	Mixed methods	Summarize multicentre PECARN implementation trial findings and reach, adoption, implementation, and maintenance of a EHR-integrated computerized tomography (CT) CDSS in the ED.	EHR based CT CDSS estimates risk of clinically important Traumatic Brain Injury (TBI) indicators and recommends CT based on the PECARN TBI prediction rules.	Physicians, nurses, assistants, and other stakeholders (*n* = 37).
Miller et al., 2019, USA [64]	Mixed methods	Describe iterative process of developing a CDSS for adolescent sexual health needs in the ED.	Decision tree facilitated as a branch-logic questionnaire using patient responses to make recommendations.	Clinicians (*n* = 57) and adolescent patients (*n* = 57).
Petitgand et al., 2020, Canada [65]	Qual	Analyze AI-based CDSS implementation in ED focusing on the actors’ representations of the system.	CDSS uses deep learning and natural language processing to triage patients presented to the ED, based on patient responses to adaptive questions.	CDSS developers, academic health center managers, ED physicians, ED nurses (*n* = 20).
Salwei et al., 2021, USA [66]	Qual	Study barriers and facilitators to workflow integration of a CDSS in ED of large academic health system.	EHR embedded CDSS combines two risk scoring algorithms recommended to support diagnosis of Pulmonary Embolism. CDSS populates data from the EHR to calculate the patient’s risk score, provides a recommendation for the next step, supports ordering the diagnostic test, and documents the decision and order in the physician’s note.	Physicians (*n* = 12).
Santucci et al., 2016, Australia [67]	Qual	Determine uptake and perceived usefulness of CDSS embedded in electronic prescribing in the ICU, and if customization is needed.	CDSS embedded within a commercial prescribing tool with alerts at point of prescribing, pre-written orders with pre-populated fields, and a reference material search tool.	Senior and junior ICU doctors (*n* = 34).
Sheehan et al., 2013, USA [68]	Qual	Describe ED sociotechnical environment to inform the design of future CDSS development for pediatric trauma.	Clinical prediction rule for children with minor blunt head trauma.	ED physicians, nursing staff and leadership, clinical IT leadership (*n* = 126); 90 h of workflow observations.
Strohm et al., 2020, the Netherlands [69]	Qual	Identify barriers and facilitators to AI implementation in clinical radiology in the Netherlands.	Commercial CDSS utilizes AI to complete automated bone maturity assessments based on X-rays of pediatric patients’ hands.	Case studies: seven hospitals. Senior and junior radiologists, legal consultant, clinical physicists, junior technical physicians, senior data scientist, managers, implementation advisors, and innovation managers (*n* = 24).
van der Stap et al., 2021, the Netherlands [70]	Qual	Evaluate feasibility and acceptability of symptom management in palliative care CDSS by exploring the views of the system’s future end users.	CDSS combines patient-reported symptom assessment scale with guideline-based recommendations and alerts clinicians to reassess symptoms.	Patient representatives, community and hospital nurses, hospital GPs, and palliative care specialists (*n* = 51).
Vandenberg et al., 2017, USA [71]	Qual	Identify facilitators and barriers to use of a CDSS in the ED to improve geriatric prescribing quality.	Prescription order entry set for geriatric patients.	ED prescribers (*n* = 20).
Weber et al., 2009, USA [72]	Qual	Explore experiences of using a CDSS for critical care including motives, and values to use or not use the technology.	Commercial CDSS uses 17 physiological variables, age, and a chronic health evaluation to predict patient outcomes.	Physicians and registered nurses (*n* = 33).
Yılmaz et al., 2017, Turkey [73]	Mixed methods	Develop and implement CDSS software for nurses working with cancer patients and explore their experiences using the CDSS.	Rule-based software providing diagnosis depending upon changes in laboratory test values and medical treatments.	Nurses (*n* = 16).
Zaidi et al., 2012, Australia [74]	Quant	Examine reliability and validity of newly developed scale measuring physicians’ perceptions of barriers and facilitators towards adoption of an antibiotic approval CDSS.	Commercial CDSS that enables approval for prescribing of restricted antibiotics and provides clinical guidelines and decision support.	Junior and senior medical staff, and pharmacists (*n* = 115).
Zaidi et al., 2013, Australia [75]	Mixed methods	Examine impact of process evaluation on uptake of web-based CDSS tool for antibiotic stewardship in a university teaching hospital.	Commercial CDSS with clinical guidelines and role-based functionality to prescribe antibiotics at the point of care.	Junior and senior medical staff, and pharmacists (*n* = 42).

*Abbreviations*: *AI* Artificial intelligence, *AMS* Antimicrobial stewardship, *CDSS* Clinical decision support system, *CPOE* Computerized provider order entry, *DVT* Deep venous thrombosis, *ECG* Electrocardiogram, *ED* Emergency department, *EHR* Electronic health record, *EMR* Electronic medical record, *GP* General practitioners, *rehab* Rehabilitation, *CDSS* Intelligent clinical decision support system, *ICU* intensive care unit, *IT* Information Technology, *SICU* Surgical intensive care unit

### Summary of facilitators and barriers to CDSS implementation (determinants)

A visual summary of the analysis, synthesis, and reporting process we undertook to move from individual barriers and enablers [A] to higher-order codes [B] and unique determinants [C] is presented in Additional file 2. In total, 227 individual barriers and 130 individual facilitators were reported across the 44 included studies (implementation determinants, *n* = 357). Sixty-five higher-order codes were deductively constructed from these barriers and facilitators during qualitative content analysis and data synthesis. Fifty of these codes (77%) could be represented as 25 matched pairs which acted as either a barrier or facilitator depending on the study and implementation context. For example, CDSS implementation was facilitated when stakeholders perceived benefits to its use but hindered when they perceived it brought little relative advantage to usual practice (determinant: perceived benefits of CDSS). Other codes were only reported to act as barriers (*n* = 12, e.g., CDSS not sensitive to complexity) or only as facilitators (*n* = 3, e.g., CDSS used in audit, feedback, and benchmarking).

**Fig. 2:**
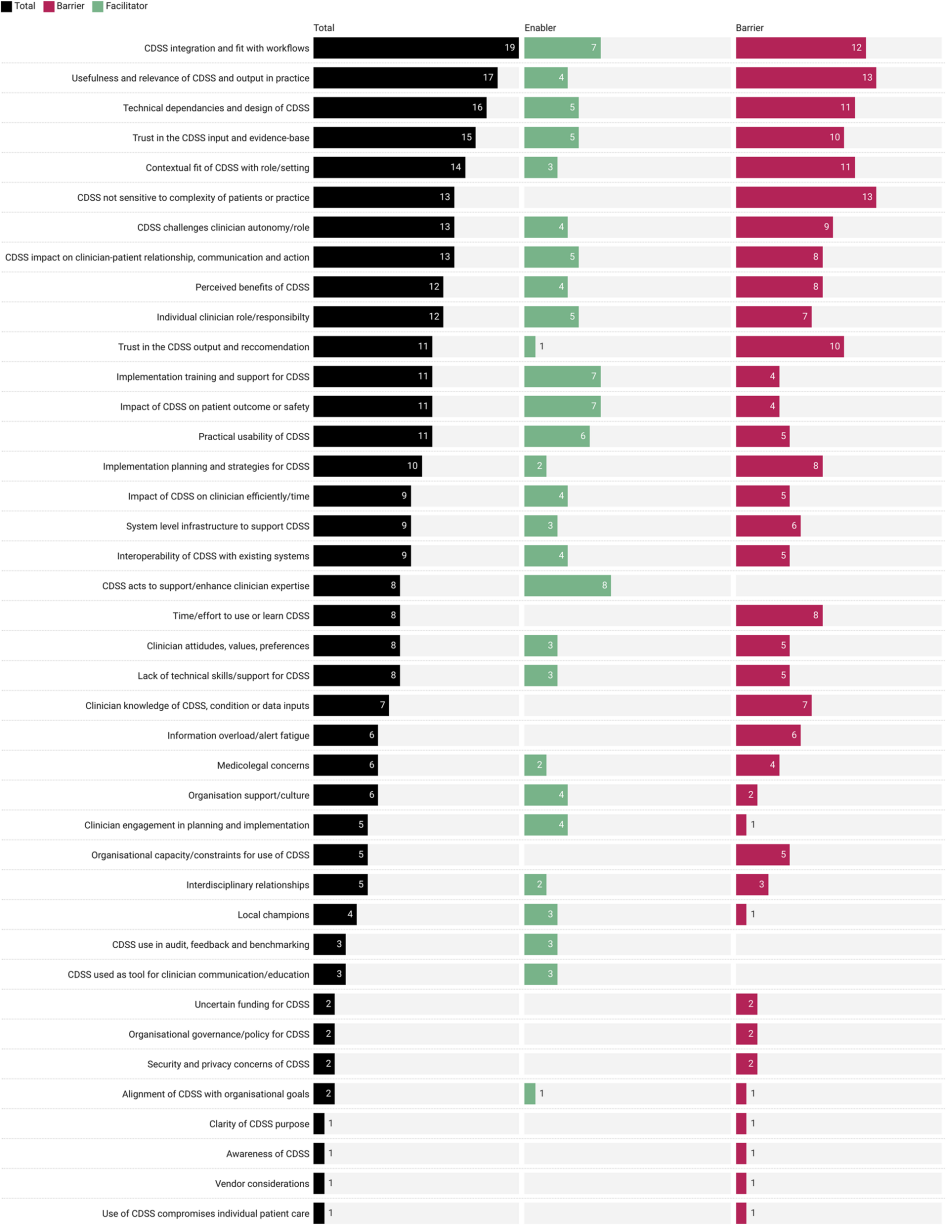
Number of included studies reporting each higher-order determinant for CDSS implementation and adoption

Combined, these higher-order codes represented 40 unique determinants influencing the implementation and use of CDSS in hospital settings (Fig. 2). The most commonly reported determinants were the fit of CDSS with workflows (*n* = 19, 44% of studies), the usefulness of the CDSS output in practice (*n* = 17, 40% of studies), CDSS technical dependencies and design (*n* = 16, 37% of studies), trust of users in the CDSS input data and evidence base (*n* = 15, 35% of studies), and the contextual fit of the CDSS with the user’s role or clinical setting (*n* = 14, 33% of studies).

**Fig. 3:**
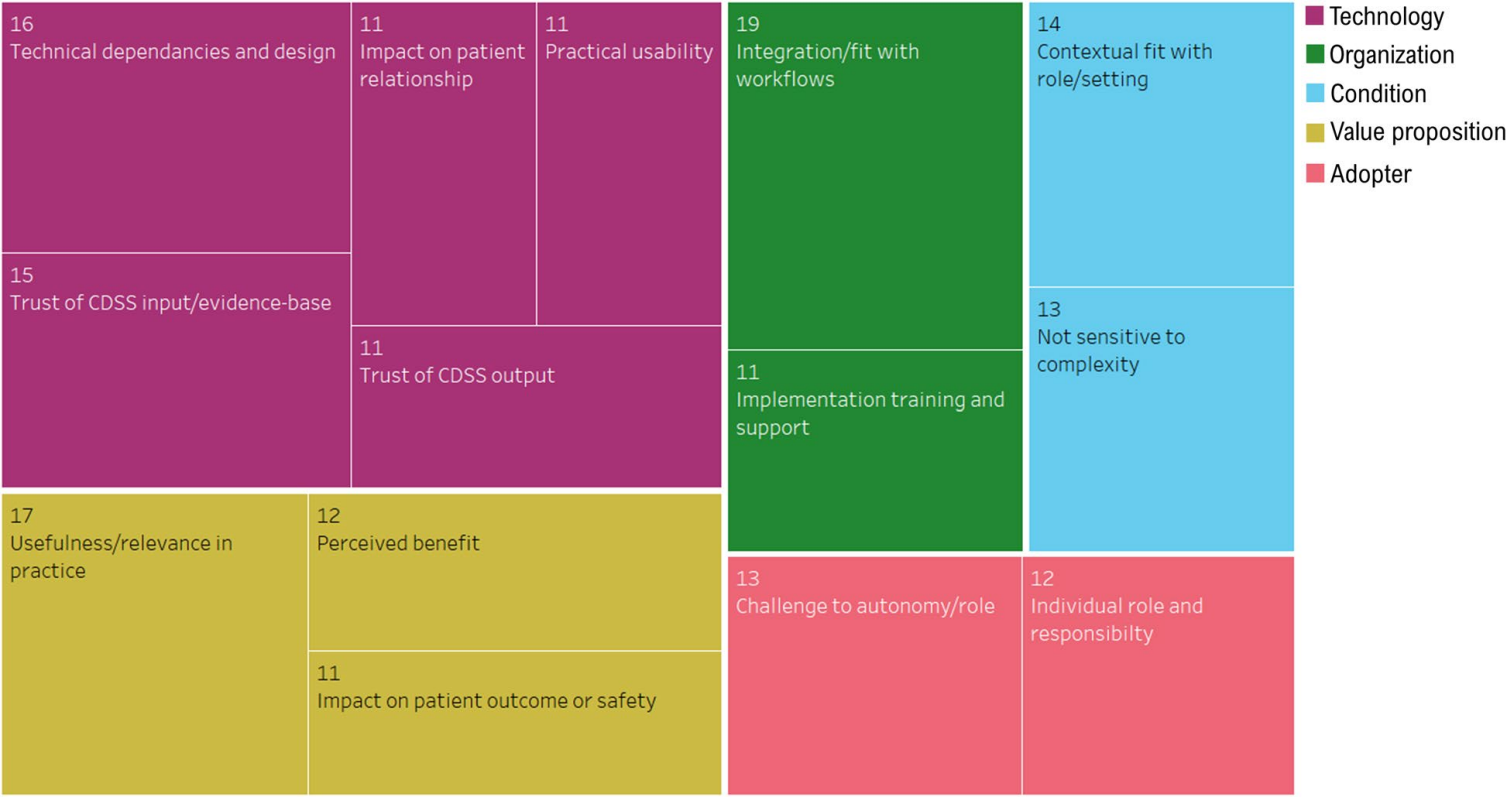
Tree map highlighting most common CDSS implementation determinants (reported in 25% or more of studies) mapped to NASSS framework domain. Numbers represent the number of studies reporting the determinant

Fourteen of these higher-level determinants were reported in 25% or more of the included studies (Fig. 3). While these common determinants occurred across almost all NASSS domains, the CDSS “technology” and “value proposition” domain contained the largest concentration of reported barriers and facilitators.

### Mapping barriers and facilitators to the NASSS framework

**Fig. 4:**
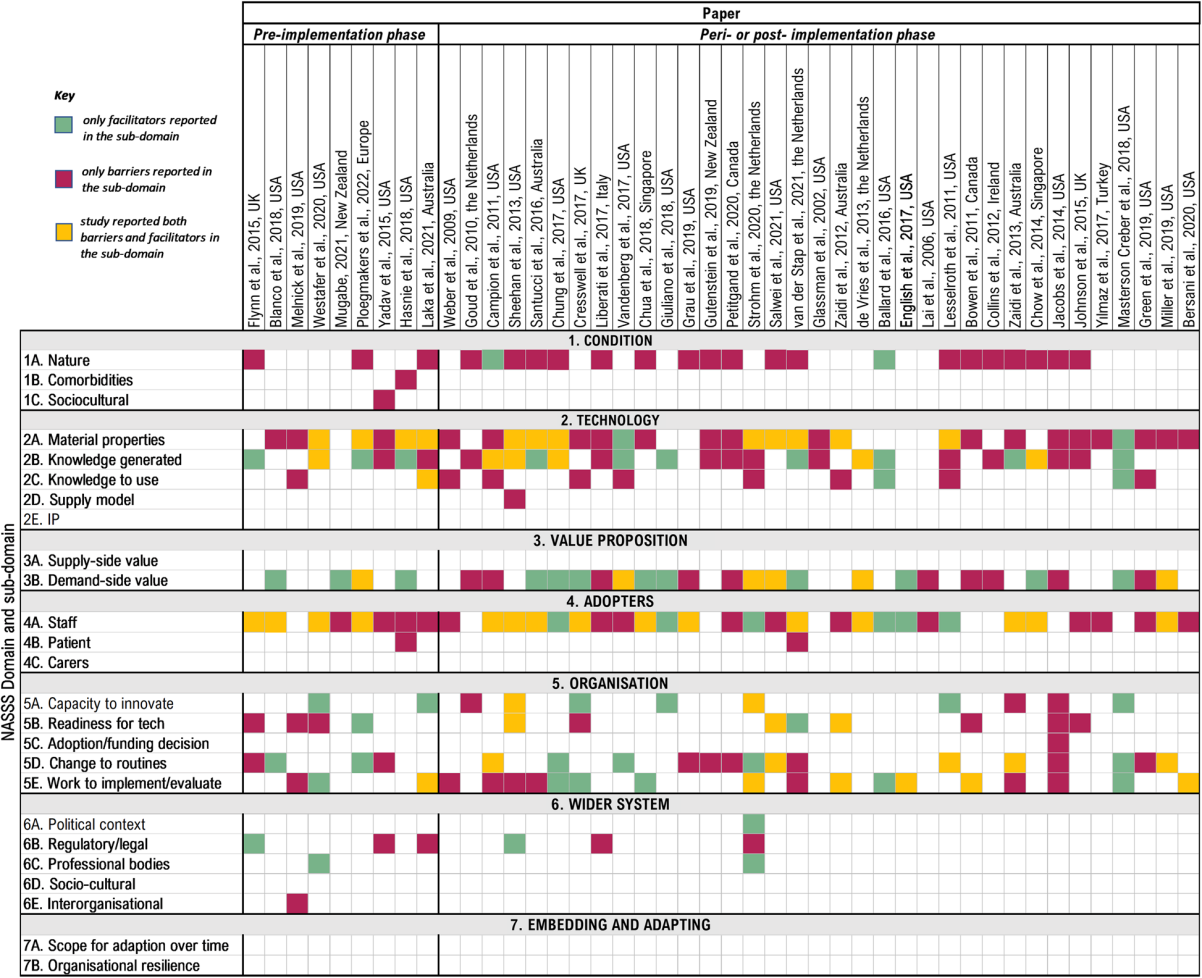
Matrix demonstrating the frequency and distribution of barriers and facilitators reported in each study across NASSS domains and sub-domains

Mapping the barriers and facilitators identified in each individual study (Additional file 2A) to one of the seven domains of the NASSS framework enabled an examination of the key areas, and potentially understudied or underreported gaps, for CDSS implementation. This is visualized as a matrix in Fig. 4. Overall, barriers and facilitators reported in individual studies were most often aligned with the NASSS domains of “Technology” (*n* = 140, 35%), “Organization” (*n* = 108, 27%), and “Adopters” (*n* = 73, 18%). No codes were assigned to the “Embedding and Adaptation Over Time” domain. More barriers than facilitators were identified related to the “Condition/Context,” “Technology,” and “Adopters.” However, for the “Value Proposition,” “Organization,” and “Wider System,” just as many facilitators as barriers were noted. A detailed summary of the barriers and facilitators related to each NASSS domain and represented in Fig. 4 is discussed below.

#### Appropriateness of NASSS framework for categorizing CDSS implementation determinants

We experienced challenges mapping some codes to suitable NASSS sub-domains. In several instances, a code was applicable to a NASSS domain but the available sub-domains were not adequate to capture the description of the barrier or facilitator. For example, in one study [65], CDSS use was hindered by having limited available service options in the community for onward patient care after a clinical decision was recommended. While this could be coded as a challenge in the “Wider System” domain, we felt it only weakly aligned with the “interorganizational networking” sub-domain we mapped it to. This was also the case for some codes aligning with the “Adopters” category, where the nuance of psychological and motivational factors related to end users was not adequately captured in the “Staff” sub-domain. Additionally, we chose to interpret the “Condition” sub-domain of “Nature of Condition” as “Clinical Context” where barriers or facilitators may have been specific to the complexity of a particular clinical area (e.g., the emergency department).

#### Condition domain

This domain refers to the contextual factors and characteristics of the healthcare condition or problem for which a particular intervention or technology is being implemented. Twenty-five studies (57%) reported at least one barrier or facilitator which could be categorized in the “Condition” domain. The condition or clinical context was most often reported to act as a barrier to CDSS implementation. The most common barriers included the *inflexibility of CDSS to allow for tailoring to contextual and situational factors* specific to a particular illness, clinical context, or patient cohort and the difficulties associated with “defining complex clinical situations within a set of rules” [50]. These were a particular concern for CDSS deployed in complex, fast-paced emergency departments [36, 65, 66, 68] for specialist clinicians, and in intensive care units [67]. Where CDSS information and recommendations could be tailored to local practice, however, implementation was facilitated.

#### Technology domain

The technology domain captured the material features, knowledge inputs and outputs of the CDSS, its procurement model, and the knowledge and skill required to use it. Almost all studies reported barriers or facilitators related to the CDSS technology itself (*n* = 40, 91%) and it consistently acted as a barrier across all sub-domains.

Many *material and technical features inherent within implemented CDSS* led to limitations and challenges in how users adopted the tools in practice. These included poor user experience, issues with the interface or physical design, alert fatigue, information redundancy, privacy concerns, technical issues, and inconsistent performance. Other issues related to the lack of CDSS integration within existing clinical pathways and limited interoperability within existing technological architecture, both requiring workarounds for successful implementation. By contrast, ensuring CDSS integrated easily into existing systems and employing user-informed design principles in its creation were both highlighted as key facilitators to implementation.

An important aspect to consider during the implementation of CDSS is the *type and quality of knowledge or information that the technology provides to end users*. Studies indicated clinicians held concerns about the completeness, quality, accuracy, and relevance of the information used to inform the decision support (e.g., evidence or guideline in knowledge-based CDSS). This often led to a mistrust of the CDSS output and lack of acceptance of the decision made. Related to this were wider issues of CDSS design such as possibilities for bias inherent in the data used (e.g., population-specific) or the level of validation required to ensure Artificial Intelligence and algorithms are sufficiently equitable. For example, consider an algorithm used to detect lung cancer from medical imaging scans. If this algorithm is trained on a dataset consisting mainly of scans from male patients, it may not perform as well when applied to scans from female patients. This gender bias in the training data can lead to disparities in the algorithm’s accuracy, potentially resulting in missed or delayed lung cancer diagnoses in women. Additionally, it is crucial to determine the appropriate level of validation needed to ensure that the integration of Artificial Intelligence and algorithms in CDSS is fair and unbiased. This validation process should address potential algorithmic biases, such as disparities in diagnostic accuracy or treatment recommendations based on race or gender. Additionally, in several studies, the CDSS output was perceived to have limited usefulness in practically delivering care. For example, one output was described as “not prescriptive enough to effectively apply to individual patients at the point-of-care” [62]. However, this technology sub-domain could equally act as an important implementation facilitator. Studies observing this sub-domain as a facilitator reported clear evidence-based inputs underpinning the CDSS to build clinician trust; a CDSS output which could be used to effectively supplement clinical expertise or judgment; and/or an output which could be used as a tool to foster communication with patients, and between teams of clinicians.

Fewer studies reported determinants related to *skills or knowledge of end users*, and most of these were barriers. Challenges were related to a lack of user knowledge of the CDSS system or clinical condition supported, coupled with limited or poor technical training and support. Perceptions that using a new CDSS would result in a loss of productivity could also deter end users from engaging with the CDSS. Two studies [62, 71] highlighted such impacts on productivity for clinicians due to the learning curve of a new technology, “*when clinical pathways were used, providers initially struggled with the order sets. Surgeons had increased order entry time and a decrease in productivity*” [62].

#### Value proposition domain

This domain explores the proposition or value that the intervention offers to users, providers, organizations, and the wider healthcare system. It is important to consider how, and for whom, a new CDSS technology generates value to ensure its successful implementation. Almost two-thirds of studies in this review (*n* = 28) reported the perceived relative advantage of using CDSS compared to usual practice was an equally important barrier and facilitator of uptake. That is, CDSS use was facilitated if it was perceived to enhance the ability of clinicians to work effectively or efficiently, improve workflow and save time, or contribute to positive patient outcomes. Additionally, if clinicians saw benefit in the role of CDSS to standardize implementation of guidelines or care, it was more readily accepted. Conversely, if the CDSS was not useful or did not deliver benefit of some kind, whether real or perceived, then it would be unlikely to be accepted. Several studies [12, 46, 50] also cited the risk of medical errors and patient safety concerns as diminishing the value of the technology.

#### Adopter system domain

The adopter system refers to the individuals, groups, or organizations that are involved in the adoption, implementation, and use of a particular innovation or intervention within the healthcare setting. Thirty-six studies (82%) reported key considerations of CDSS adoption related to its users. Determinants in this domain more often acted as barriers than facilitators; however, some key enabling factors were noted. While the NASSS framework considers staff, patients, and caregivers in this domain, the studies in this review almost exclusively focused on healthcare professionals. The key themes related to perceptions of impact of CDSS on their role, responsibility, and professional autonomy; personal factors such as general attitudes and motivations; and their physical capability and opportunity to use the CDSS. Only two papers [37, 70] highlighted barriers which were perceived to impact patient adoption of CDSS. The main concerns were the requirement of patients to enter data into the system, and patients who did not act on recommendations provided by the clinician and CDSS.

Staff perceptions of professional autonomy were perceived to be either reduced or enhanced by CDSS. This depended on whether CDSS was seen as a tool that inhibited or facilitated clinician ability to make judgments. While implementation and adoption were hindered when clinicians had “fear of losing control over autonomous decision-making” [12], where local implementation allowed professional autonomy to be balanced with the standardization provided by the CDSS output, adoption was facilitated. Similarly, perceptions of professional role identity and responsibility were both positive and negative influences on implementation. CDSS were seen as enabling professionals of different specialties to engage in the decision-making domains of others. This was seen as a barrier in some specialties where there may be clearly defined hierarchies, but as a facilitator where CDSS could reinforce the tasks and responsibilities of one particular profession (e.g., Doctor) compared to another (e.g., Pharmacist).

Perceptions, irrespective of their accuracy, about the time and effort required to use a CDSS, its complexity, availability, usability, timeliness, or usefulness in particular work contexts were reported to reduce user motivation for CDSS. Lack of acceptance, intentional passive resistance, and cognitive influences (e.g., memory and attention) often meant users reverted to workflows and processes they were more comfortable with. Psychological characteristics of individual end users also emerged as barriers to the adoption and implementation of CDSS. This included individual preferences and attitudes about the features, usability, or perceived benefits of a CDSS. The level of risk tolerance exhibited by individual clinicians can also influenced their willingness to trust and therefore use CDSS. Perceptions about CDSS being less accurate also led to user hesitancy among clinicians. Additionally, the perceived relationship with the patient also adversely impacted uptake of CDSS. Healthcare professionals who highly value personal interactions and direct patient engagement harbored concerns about the potential disruption of the doctor-patient relationship that may arise with the use of technology-driven interventions.

Finally, although reported to a lesser extent, capability barriers incorporated issues such as lack of knowledge, skills, or familiarity with the CDSS. Physical opportunity barriers related to limited time to invest in learning and using the new CDSS.

#### Organization domain

This domain considered organizational capacity and readiness to implement CDSS, funding and costs of the new technology, implementation processes, and changes to team interactions and routines. Interestingly, the 35 studies (80%) categorized in this domain reported barriers and facilitators to occur almost equally.

One of the most frequently reported considerations in the organizational domain was the *extent of change needed to organizational routines to implement a new CDSS*. More likely to act as a barrier in included studies, this was reflective of CDSS fit with existing workflows, and existing governance practices. CDSS implementation that required minimal workflow changes, interruptions, or unnecessary duplication of activities were more likely to be successful.

An *early, ongoing, and supported implementation plan* was also reported as one of the most frequent facilitators of CDSS implementation in the organization domain. Components of successful implementation strategies included local champions and super-users that promoted and supported use of the CDSS; benchmarking, audit, and feedback to drive change; provision of technical training and support; and early engagement and involvement of users in the development and implementation of the CDSS. Conversely, a lack of planned implementation process was reported by other studies to lead to poor organizational preparedness and limited buy-in which hindered implementation.

Organizations that demonstrated a *good overall capacity to innovate* provided enabling environments for CDSS implementation. This incorporated a positive institutional culture, clear innovation strategy, organizational support for change, and previous successful technological deployment. Conversely, limited leadership buy-in for innovation or a lack of organizational policies or goals to innovate created barriers for CDSS implementation. Some organizations were reported to have an ingrained culture that was generally resistant to change. Thus, the successful introduction of an innovation such as CDSS was unlikely to be successful unless this cultural factor was addressed. The *readiness of the organization for technology-supported change* was more often reported to be a barrier in included studies, highlighting such concerns as limitations in the system-level infrastructure or information technology resources to enable CDSS implementation. Similarly, issues related to governance represented organizational barriers with respect to the readiness of policies, procedures, and reporting requirements for CDSS. Only one paper [59] specifically reported that consideration of *increased costs* of resources for the organization under conditions of uncertain funding could be a barrier to CDSS use.

#### Wider system domain

The wider system domain considers factors such as the political, economic, and social contexts within which the healthcare system operates. Only eight papers (18%) reported determinants of CDSS implementation which sat in the wider healthcare, social, or policy system. The majority of these (*n* = 6) were concerned with *regulatory or medico-legal issues* related to aspects of CDSS clinical use or compliance. For example, in two studies [12, 69], clinicians were concerned about where legal responsibility would lie if patient harm occurred when following CDSS recommendations. However, one study [68] reported CDSS would have better adoption if it provided data to help meet external regulatory requirements. Two studies [41, 69] reported on the *enabling nature of professional bodies in CDSS implementation*. This was because they could provide guidelines to support CDSS input, provide a platform for knowledge exchange, or lead an implementation agenda to increase use in different contexts and professions. Finally, *pressure on healthcare budgets* was identified as a facilitator for the adoption of CDSS in one paper [69] when there was an expectation that use of such technologies would lead to efficiencies or increased effectiveness and therefore reductions in costs.

## Discussion

Our study identified a comprehensive set of determinants prioritized by the literature that impact the implementation process of CDSS within hospital systems. These were captured across a mix of study designs, geographic locations, participants, technology types, CDSS functions, and clinical contexts of implementation. In doing so we have systematically identified common barriers and facilitators which can inform the design of targeted implementation strategies to enhance the adoption of CDSS within hospitals. In particular, the findings suggest that there is a need for CDSS to be designed to fit the workflows and contexts of clinical practice and that enhancing user trust in the accuracy, value, and relevance of the CDSS and its output is crucial for effective implementation outcomes. This finding is supported by previous literature which has emphasized the importance of implementation context and attaining good “clinician-patient-system integration” when developing CDSSs [76]. This review highlights the importance of further integrating implementation science principles into CDSS intervention design [6].

Adopting a theory-informed implementation science approach to this review has also enabled an examination of the complexity of CDSS implementation in real-world healthcare settings. To our knowledge, this is the first review to systematically identify, classify, and map peer-reviewed literature about barriers and facilitators of hospital-based CDSS implementation using an implementation science framework. Two recent systematic reviews assessing the determinants of CDSS uptake have been limited to relatively linear descriptions or analysis of the issue (i.e., including a recent pooled analysis with extreme heterogeneity [6, 77]). This underscores the value of a framework-informed classification of these determinants which identifies multiple implementation influencers along the adoption-user-sustainment pathway to inform a cascade of targeted implementation strategies.

By addressing the common barriers and facilitators identified, stakeholders can employ context-relevant implementation strategies to more effectively promote and sustain CDSS implementation outcomes [78]. To achieve this, the authors recommend decision makers should involve end users in the design and testing phases of CDSS to ensure that the system meets contextual needs and is aligned with existing workflows. This may allow for more effective and accurate resource prioritization [13]. Transparency and systematic governance of CDSS may build trust in the data sources and the accuracy of outputs, ensuring the CDSS is being used appropriately and in the best interests of patients [8]. Providing ongoing support and training to end users can address perceptual and psychosocial determinants of CDSS acceptance, enabling users to effectively use the system [10, 11]. Tailoring implementation strategies to different service contexts may address organizational-level determinants related to workflow integration and ensure the CDSS is compatible with the hospital’s existing processes and infrastructure [7]. Advocating for legislative clarity regulating the use of CDSS for clinician decision-making can potentially address wider system barriers, providing clarity, and may boost confidence in the use of CDSS tools.

Our review supports the value of the NASSS framework for assessing context and guiding CDSS implementation. It offers a nuanced view of determinants, processes, and outcomes by considering the dynamic interplay between the system, the context, and the stakeholders. In our review, most determinants, with very few exceptions, could be appropriately categorized by the contextually specific domains within the NASSS framework. This illustrates the external validity of this framework to identify and meaningfully categorize CDSS implementation determinants within hospital settings. Similar validity of the NASSS framework has been observed in reviews examining the implementation of artificial intelligence in health care settings [76] and virtual care implementation within primary care [22]. However, as was the case in our mapping, the authors of that paper also experienced challenges aligning some of the determinants with existing NASSS sub-domains.

Consequently, these findings suggest that NASSS may need to be further contextualized to account for individual technologies. Greater clarification of existing domains and sub-domains is required to understand how implementation of different technologies may be categorized within the existing framework. For example, understanding whether the adaptations to “The Condition” we made in this review are consistent with the underlying theory of NASSS. Additionally, new evidence-based sub-domains (for example in “The Adopters” or “Wider Condition”) may be required to supplement the current NASSS framework. With such refinements, NASSS would have better utility for context assessment and development of targeted implementation strategies to enhance the adoption, integration, and sustainability of CDSS in hospitals.

Additionally, a noteworthy limitation of the literature identified is the lack of identified determinants mapped to the “embedding over time” domain of NASSS. This reflects an ongoing need to systematically evaluate and document how adoption factors around digital health interventions are adapted and changed over time. This could be partially due to the limited funding opportunities available for longitudinal studies that extend beyond the initial evaluation phase. Understanding these determinants are especially important for acute health services like hospitals which face dynamical and changing contextual pressures related to budgets, socio-political events, and natural disasters. Furthermore, we note that most studies focusing on healthcare stakeholders predominantly included frontline clinicians and other health workers. We recommend future work also include other informants involved in policy, informatics, and health management which may serve to unpack wider-domain factors with greater specificity.

Limitations of this review are excluding studies which only described intervention development or conducted context assessments without any explicit reference to a CDSS, which might adjust for contextual determinants during the intervention development process (informed by context assessments and related co-design strategies). Furthermore, while patient-reported factors were not explicitly excluded from this review, very few (*n* = 2) included studies reported on this perspective. These excluded perspectives may serve to further contextualize and potentially add to the determinants identified. It is currently unclear how and if health consumer perspectives are generally sought during the intervention roll-out phases of CDSS within hospitals, and this constitutes a significant gap in our knowledge of these potentially important determinants. It must also be noted that the reported health system determinants of CDSS adoption do not represent an exhaustive list, since the absence of evidence does not indicate evidence of absence.

## Conclusions

Ongoing efforts are needed to categorize common factors within hospital systems that influence the adoption, integration, and sustainability of digital health solutions. This will enable the optimal integration of digital innovations into hospital settings, ensuring intended improvements in outcomes can be achieved and maintained. Applying established implementation science approaches, such as using theoretical frameworks and models, can enhance the efficiency and effectiveness of this process in a systematic and contextually appropriate manner. The findings of our review contribute to assisting healthcare stakeholders in identifying key determinants that can be modified to facilitate the adoption of Clinical Decision Support Systems (CDSS) in various hospital settings.

## Supplementary Information

**Additional file 1.** Refinement of search strategy.

**Additional file 2.** Visual summary of synthesis and analysis process and mapping to key results and figures.

## Data Availability

The authors declare that the data supporting findings of this review are available within the paper.

## Abbreviations

CDSS: Computerized clinical decision support system
NASSS: Nonadoption, Abandonment, Scale-up, Spread, and Sustainability
