# Supplementary material for: Identifying barriers and facilitators to successful implementation of computerized clinical decision support systems in hospitals: a NASSS framework-informed scoping review
Source: Implement Sci. 2023 Jul 26;18:32. doi: 10.1186/s13012-023-01287-y (PMC10373265; doi:10.1186/s13012-023-01287-y)
Supplement: Supplementary file 1 — Additional file 1. Refinement of search strategy. [file 13012_2023_1287_MOESM1_ESM.docx]

# Additional file 1. Refinement of search strategy

## Original/pilot strategy

Prior to the first database search an original Boolean search string consisting of 30 search terms was generated and a preliminary search conducted in Ovid MEDLINE.

1. exp Decision Support Systems, Clinical/
2. exp Medical Records Systems, Computerized/
3. exp Artificial Intelligence/
4. exp Decision Support Techniques/
5. exp Decision Making, Computer-Assisted/
6. Reminder Systems/
7. "decision support system*"
8. "Computeri?ed reminder*"
9. "Machine learning"
10. Dashboard
11. "Reminder system*"
12. "Computer* feedback"
13. "electronic feedback"
14. CDSS
15. OR 1-14
16. exp Diffusion of Innovation/
17. disseminat*
18. implement*
19. Barrier*
20. Challeng*
21. Facilitat*
22. Enable*
23. Factor*
24. Percept*
25. Attitude*
26. OR 16-25
27. exp Hospitals/
28. exp Hospital Units/
29. exp Hospital Departments/
30. emergency department
31. outpatient
32. inpatient
33. OR 27-32

15 AND 26 AND 33

## Refinement of strategy

## Visualisation and refinement of strategy with searchrefiner tool (<https://ielab.io/searchrefiner/>) to highlight key terms and eliminate noise using PMIDS of relevant articles returned via the original search and a complementary Google Scholar search:

## 2353276

## 19159458

## 15135754

## 25163794

## 26459233

## 28915822

## 23622342

## 25434998

## 29477289

## 28926204

*Final strategy after refinement*

1. Exp/ Decision Support Systems, Clinical
2. "Artificial Intelligence"[Title/Abstract]
3. "Reminder system*"[Title/Abstract]
4. "electronic feedback"[Title/Abstract]
5. CDSS[Title/Abstract]
6. OR 1-5
7. Barrier*[Title/Abstract]
8. Facilitat*[Title/Abstract]
9. OR 7-8
10. 6 AND 9

These terms were used in the search strings for each database for both the first search (July 2020) and the follow up search (May 2022). The MEDLINE search was copied into the Polyglot Search Translator (<https://sr-accelerator.com>) which automatically translated the search across multiple databases by modifying the required syntax. Each search string was then run in the relevant database and iteratively optimised for that database. For example, to ensure consistency in subject headings or capture additional relevant subject headings across databases as recommended by Kung JY, 2022 (1).

**Table A1*.*** Databases and final search strings used to identify articles for the study

| Database | Search string |
| --- | --- |
| MEDLINE (Ovid) | ((exp Decision Support Systems, Clinical/ OR Artificial Intelligence.ti,ab. OR Dashboard.ti,ab. OR Reminder system*.ti,ab. OR electronic feedback.ti,ab. OR CDSS.ti,ab.) AND (barrier*.ti,ab. OR Facilitat*.ti,ab.)) |
| Embase | ((‘Decision Support Systems, Clinical’/exp OR “Artificial Intelligence”:ti,ab OR Dashboard:ti,ab OR “Reminder system*”:ti,ab OR “electronic feedback”:ti,ab OR CDSS:ti,ab) AND (barrier*:ti,ab OR Facilitat*:ti,ab)) |
| CINAHL | (((MH “Decision Support Systems, Clinical+”) OR TI “Artificial Intelligence” OR AB “Artificial Intelligence” OR TI Dashboard OR AB Dashboard OR TI “Reminder system*” OR AB “Reminder system*” OR TI “electronic feedback” OR AB “electronic feedback” OR TI CDSS OR AB CDSS) AND (TI barrier* OR AB barrier* OR TI Facilitat* OR AB Facilitat*)) |
| PsychINFO | ((DE” Decision Support Systems” OR “Artificial Intelligence”.ti,ab OR Dashboard.ti,ab OR “Reminder system*”.ti,ab OR “electronic feedback”.ti,ab OR CDSS.ti,ab) AND (barrier*.ti,ab OR Facilitat*.ti,ab)) |
| Scopus | ((INDEXTERMS(“Decision Support Systems, Clinical”) OR TITLE-ABS(“Artificial Intelligence”) OR TITLE-ABS(“Dashboard”) OR TITLE-ABS(“Reminder system*”) OR TITLE-ABS(“electronic feedback”) OR TITLE-ABS(“CDSS”)) AND (TITLE-ABS(“barrier*”) OR TITLE-ABS(“Facilitat*”))) |

Note. Embase search excluded those indexed in MEDLINE; Scopus search excluded those indexed in MEDLINE and Embase; PsychINFO mesh heading “Decision Support Systems’ was changed/updated in the database between the two searches.

1. Kung JY. Polyglot Search Translator. The Journal of the Canadian Health Libraries Association. 2022 Apr;43(1):35
