# Supplementary material for: Identifying barriers and facilitators to successful implementation of computerized clinical decision support systems in hospitals: a NASSS framework-informed scoping review
Source: Implement Sci. 2023 Jul 26;18:32. doi: 10.1186/s13012-023-01287-y (PMC10373265; doi:10.1186/s13012-023-01287-y)

Additional file 2. Visual summary of synthesis and analysis process and mapping to key results and figures

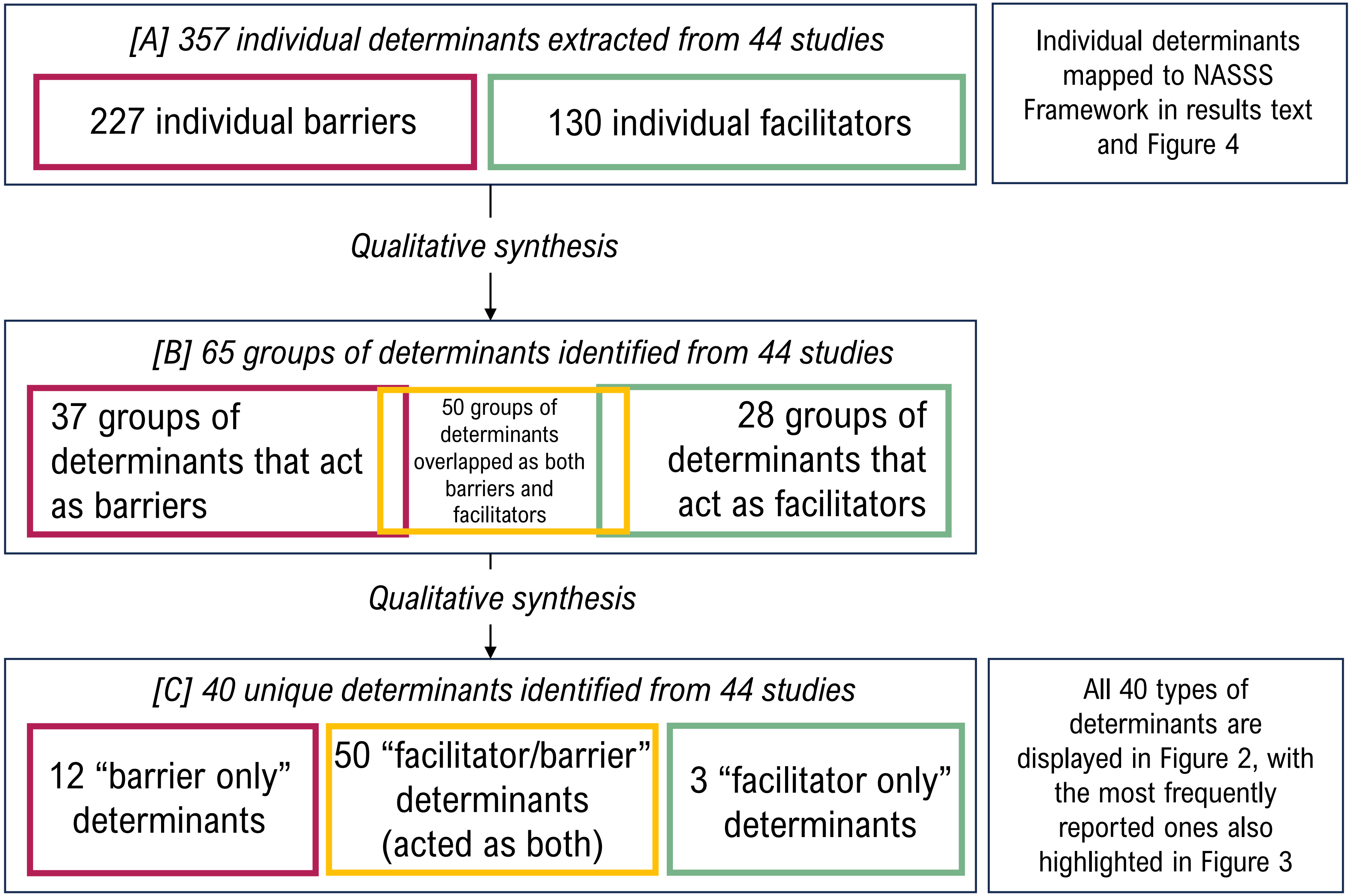

Supplement: Supplementary file 2 — Additional file 2. Visual summary of synthesis and analysis process and mapping to key results and figures. [file 13012_2023_1287_MOESM2_ESM.pdf]
